# An examination of the relationship between perceptions that cigarette smoking increases the severity of COVID-19 and reduction in smoking during the COVID-19 pandemic: Findings from the 2021 ITC Korea Survey

**DOI:** 10.18332/tid/205468

**Published:** 2025-06-30

**Authors:** Hae-ryoung Chun, Geoffrey T. Fong, Shannon Gravely, Steve S. Xu, Anne C. K. Quah, Heewon Kang, Wonjeong Yoon, Hong G. Seo, Yeol Kim, Sungkyu Lee, Gil-yong Kim, Chang Bum Kang, Sujin Lim, Mi Yan, Sung-il Cho

**Affiliations:** 1 Department of Psychiatry, College of Medicine, The Catholic University of Korea, Seoul, Korea; 2 Department of Public Health Sciences, Graduate School of Public Health, Seoul National University, Seoul, Republic of Korea; 3 Department of Psychology, University of Waterloo, Ontario, Canada; 4 School of Public Health Sciences, University of Waterloo, Ontario, Canada; 5 Ontario Institute for Cancer Research, Toronto, Ontario, Canada; 6 Institute of Health and Environment, Seoul National University, Seoul, Republic of Korea; 7 NHMRC Centre of Research Excellence on Achieving the Tobacco Endgame, School of Public Health, The University of Queensland, Brisbane, Queensland, Australia; 8 Department of Family Medicine, National Cancer Center, Goyang-si, Republic of Korea; 9 Graduate School of Cancer Science and Policy, National Cancer Center, Goyang-si, Republic of Korea; 10 Korea Center for Tobacco Control Research and Education, Seoul, Republic of Korea; 11 National Tobacco Control Center, Korean Health Promotion Institute, Seoul, Republic of Korea

**Keywords:** COVID-19, cigarettes, perceived risk, health behavior change, Republic of Korea

## Abstract

**INTRODUCTION:**

This study examined whether Korean adults who smoked cigarettes during the COVID-19 pandemic decreased their smoking because of COVID-19, with a focus on whether perceptions of susceptibility and severity of COVID-19 disease were associated with reduced smoking.

**METHODS:**

Data came from 1506 Korean adults (aged ≥19 years) who exclusively smoked cigarettes (weekly) and participated in the 2021 International Tobacco Control (ITC) Korea Survey. Weighted analyses assessed pandemic-related changes in smoking and whether reductions in smoking were related to: 1) perceived susceptibility to contracting COVID-19; 2) perceiving that smoking increases the severity of COVID-19 disease; and 3) general worry about getting a smoking-related disease.

**RESULTS:**

Korean adults were significantly more likely to have reduced their smoking because of COVID-19 (18.9%) than increased their smoking (6.3%) (p<0.001); 74.8% made no changes to their smoking. Reducing smoking was associated with being very worried about contracting COVID-19 (adjusted odds ratio, AOR=4.25; 95% CI: 1.65–10.99) and believing that smoking increases COVID-19 severity (AOR=2.34; 95% CI: 1.19–4.61). General worry about getting smoking-related diseases was not associated with smoking reduction (p=0.53). We also found an interaction between perceived severity and worry about getting COVID-19; those who were very worried about getting COVID-19 and perceive smoking as increasing its severity, were more likely to reduce smoking (p=0.049).

**CONCLUSIONS:**

In response to the COVID-19 pandemic, Korean adults who smoked were much more likely to reduce than increase their smoking, unlike in most countries where there was no net change. The higher smoking reduction rate in Korea may reflect successful and strong communication by the Korean government regarding the importance of reducing smoking during the pandemic, compared to the general future threat of health risks from smoking.

## INTRODUCTION

Tobacco smoking is a known risk factor for many respiratory infections^1^. In April 2020, very early in the COVID-19 pandemic, the World Health Organization (WHO) declared that people who smoke were more likely to develop more severe COVID-19 compared with people who have never smoked, emphasizing the urgent need for cessation^2^. Given the connection between smoking and severity of COVID-19 illness that were communicated by public health authorities, there have been a number of studies that have assessed the impact of the COVID-19 pandemic on smoking.

Past studies of the impact of major negative population events on smoking like 9/11 or earthquakes, have found that they have increased smoking, which has been attributed to psychosocial stress, negative affect^3^ and posttraumatic stress disorder^3^. In response to the COVID-19 pandemic, however, although smoking was found to increase in a previous study conducted in Poland^4^, no net change in smoking was found in other studies conducted in Canada^5^, US^5,6^, England^5^, and Australia^5^. In an earlier review on changes in smoking frequency after the COVID-19 pandemic, some studies reported a decrease, while others reported an increase^7^. Another review found that most studies observed a decrease in the prevalence of smoking after the pandemic compared to before, although some studies did report an increase^8^. Overall, however, the COVID-19 pandemic did not seem to uniformly increase smoking, in contrast to other negative population-level events, which have generally led to increased smoking.

One possible explanation for why the COVID-19 pandemic may have differed from natural disasters in not leading to increases in smoking is that unlike natural disasters, where smoking had not relationship to the disaster, COVID-19 illness is a serious respiratory disease and as such is linked to smoking. In response to public health messages that smoking is related to severity of COVID-19 illness, those who smoke would have greater incentive to quit or reduce their smoking, believing that doing so would reduce their likelihood of infection and the severity of their illness if infected^5^. This account highlights the importance of perceptions of susceptibility and illness severity in understanding how the COVID-19 pandemic affected smoking.

This study aims to analyze the proportion of respondents who reported changes in smoking due to COVID-19 in the Republic of Korea. There is reason to believe that in Korea, unlike many other countries, conditions would be more favorable for the pandemic to lead to reduced smoking. In Korea, the government response to the pandemic was strong and decisive, and very well supported by the public. Instead of aggressive measures like immigration control, lockdowns, or roadblocks, Korea focused on tracing, testing, and treating^9^. The experience of numerous fatalities during the Middle East respiratory syndrome coronavirus outbreak in 2015 likely spurred early and proactive responses to the COVID-19 pandemic aimed at preventing similar outcomes^10^. Koreans developed a deep understanding of the collective action to prevent widespread infection^10^. From the onset of the pandemic, mask-wearing, one of the most effective COVID-19 preventive measures^11^, was mandatory in Korea. An international survey showed that 94% of Koreans wore masks, the highest rate among 28 countries surveyed^12^. This was in contrast to other countries where health authorities did not recommend mask-wearing unless people were symptomatic due to a lack of evidence on efficacy^13^. Koreans thus trusted and were more likely to follow the government’s recommendations regarding reducing risk of COVID-19, including the government’s communications (frequent messaging and a well-circulated infographic on the link between smoking and the severity of COVID-19^14^). We thus predicted that in Korea, COVID-19 would have led to significant reductions in smoking.

A second focus of the present study was to test the extent to which two psychosocial variables from the Health Belief Model (HBM) – perceived susceptibility and disease severity beliefs – were associated with reducing smoking due to COVID-19^5,15^. The declaration of the COVID-19 pandemic and the classification of people who smoke as a high-risk group constitute external cues that may alter perceptions of disease severity and susceptibility, potentially motivating people to reduce smoking. A multi-country study found that adults who smoked in Australia, Canada, England, and the US were more likely to try quitting or reducing smoking if they had significant worries about COVID-19 susceptibility and severity^5^. In May 2020, a US study found that knowledge of smoking increasing COVID-19 risk was associated with increased interest in quitting^16^. Similarly, a survey in the United Kingdom showed that the perceived likelihood of contracting COVID-19 mediated the positive impact of COVID-19 fear on smoking cessation motivation^17^. Additionally, a study of adults who did and did not smoke in Ohio (US) used the HBM framework to develop a COVID-19-related risk perception questionnaire and found that for people who smoke, the desire to quit smoking since the outbreak was associated with the belief that they were personally at risk of severe COVID-19 infection^18^. However, these studies were conducted in early 2020 when COVID-19 had just emerged. They also did not consider people who smoke only cigarettes (e.g. they may have used other nicotine products)^16-18^; therefore, people who use multiple nicotine products might decrease their cigarette consumption while increasing their use of the other nicotine product. This may not reduce their overall nicotine intake and could inaccurately reflect changes in cigarette smoking.

Few studies have examined the impact of the COVID-19 pandemic on smoking in Korea. One study found that higher household income, temporary worker status, living with others, and fewer offline friends were associated with decreased smoking^19^. However, participants in that study also included people who dual used cigarettes and other nicotine products (e.g. e-cigarettes)^19^. The inclusion of those who used cigarettes and other nicotine products creates challenges in interpreting the results since the observed reduction in cigarette consumption may have been due to increased use of other nicotine products. Considering the well-known health risks associated with cigarette smoking, investigating the decrease in smoking among people who exclusively smoked cigarettes during the COVID-19 pandemic is necessary to guide public health strategies.

Using a nationally representative survey in Korea, we aimed to assess the proportion of adults who reduced smoking during the pandemic. We also sought to determine whether perceived susceptibility to COVID-19, the belief that smoking worsens COVID-19 severity, and general worry about smoking-related disease influenced smoking reduction during the second year (2021) of the COVID-19 pandemic.

## METHODS

### Data and study sample

Data were from the Wave 2 International Tobacco Control Korea (ITC KRA2) Survey conducted from 3 November to 13 December 2021. The KRA2 sample included Korean adults (aged ≥19 years) who were using nicotine products (smoked cigarettes or were using nicotine vaping products and/or heated tobacco products at least weekly) or those who either formerly or never used them. A total of 4467 respondents were sampled through Rakuten Insight’s web panel, with sample weights constructed based on age, sex, and geographical region to be representative of the Korean population^20,21^.

For the current study, the sample was restricted to 1825 respondents who had smoked at least 100 cigarettes in their lifetime and currently smoked only cigarettes at least weekly. Respondents who smoked at least weekly and concurrently used other nicotine products were excluded to avoid confusion regarding whether reductions in smoking cigarettes coincided with the use of other nicotine products. Among them, 319 were excluded due to missing values for the response or explanatory variables, resulting in a final sample of 1506 respondents in the logistic regression analysis. Supplementary file Figure 1 provides a flow diagram of the study’s selection of eligible participants.

### Study framework

**Figure 1:**
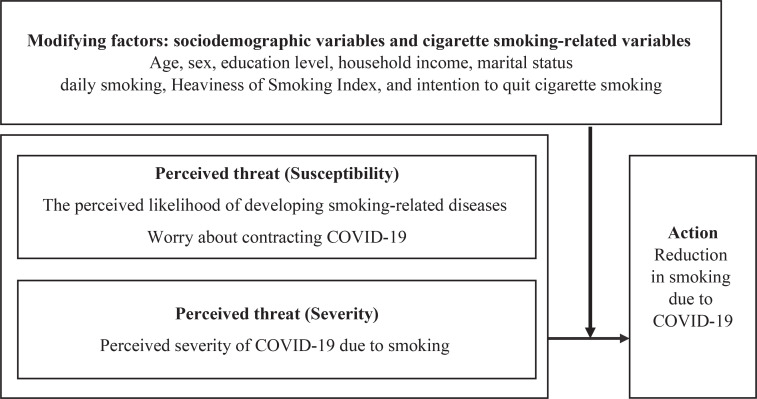
Adapted framework based on the Health Belief Model (HBM), illustrating the hypothesized association between COVID-19 risk perceptions and smoking reduction among Korean adults (2021 ITC Korea Survey)

Like the previous study^18^, we examined the effects of perceived susceptibility and severity elements of the HBM, widely used to predict changes in health behavior^22^, on reducing smoking, as presented in Figure 1.

### Modifying factors

*Sociodemographic variables*

The analysis incorporated six demographic characteristics: sex, age group (19–29, 30–39, 40–59, ≥60 years), education level, annual household income, and marital status.

Education level

Respondents were asked: ‘What is the highest level of formal education that you have completed?’. Those who answered, ‘Primary school’, ‘Middle school’, or ‘Completed high school’ were classified as low level (high school or below). Those who selected ‘Some university, no degree’ or ‘Completed university degree’ were classified as middle level (some college). Those who reported, ‘Postgraduate degree’ were classified as high level (graduate school or higher). Respondents who selected ‘Never attended school or dropped out of school’, ‘Other’, ‘Refused’, or ‘Don’t know’ were excluded from the analysis.

Annual household income

Respondents were asked: ‘What is your annual household income?’. Those who answered ‘<30 million KRW’ were classified as low-income, those who reported ‘30 million to less than 75 million KRW’ were classified as middle-income, and those who reported ‘≥75 million KRW’ were classified as high-income. Respondents who selected ‘Refused’ or ‘Don’t know’ were excluded from the analysis.

Marital status

Respondents were asked: ‘What is your marital status?’. Those who selected ‘Married’ or ‘Common-law’ were categorized as married, and those who selected ‘Single’ were categorized as unmarried. Respondents who selected ‘Separated’, ‘Divorced’, ‘Widowed’, ‘Refused’, or ‘Don’t know’ were excluded from the analysis.

*Smoking-related variables*

Nicotine dependence

The Heaviness of Smoking Index (HSI) was determined by two questions. First, ‘How soon after waking do you usually have your first cigarettes?’ with scoring as follows: ‘≤5 min’ (3 points), ‘6–30 min’ (2 points), ‘31–60 min’ (1 point), ‘After 60 min’ (0 points). The second question was, ‘On average, how many cigarettes do you smoke each day’ with scoring as follows: ‘≤10’ (0 points), ‘11–20’ (1 point), ‘21–30’ (2 points), ‘≥31’ (3 points). The cumulative score from both questions ranged from 0 to 6. This analysis further categorized nicotine dependence levels into 0–1 points (low), 2–4 points (moderate), and 5–6 points (high).

Intentions to quit smoking

All respondents were asked: ‘Are you planning to quit smoking?’. Those who answered, ‘Within the next month’, ‘Between 1 and 6 months from now’, and ‘Sometime in the future, beyond six months’ were grouped as ‘yes’ due to the small sample sizes for each category. Those responding, ‘Not planning to quit’ or ‘Don’t know’ were classified as ‘no’. Respondents who selected ‘Refused’ or ‘Don’t know’ were excluded from the analysis.

*Threat variables: perceived susceptibility and severity*

Susceptibility

To assess general worry about getting a smoking-related disease, respondents were asked: ‘If you continue to smoke as much as you do now, compared to people who previously smoked or report not smoking for the past year, what are the chances that you will develop a smoking-related disease, such as lung cancer, heart disease, or emphysema?’. Options included: ‘Much more likely’, ‘Somewhat more likely’, ‘A little more likely’, ‘Just as likely’, ‘Less likely’, ‘Don’t know’, and ‘Refused’. Those who answered: ‘Much more likely’ or ‘Somewhat more likely’ were combined into ‘Much more/somewhat more likely’, and those who answered ‘A little more likely’ were classified as ‘A little more likely’. Respondents who answered ‘Just as likely’, ‘Less likely’, or ‘Don’t know’ were classified as ‘Less likely’. ‘Refused’ responses were excluded from the analysis.

A more specific immediate threat to health was assessed by asking respondents: ‘How worried are you that you already have or will contract the coronavirus?’. Those who answered ‘Extremely worried’ or ‘Very worried’ were combined into one response. Those who answered ‘A little worried’ or ‘Somewhat worried’ were combined into ‘A little or somewhat worried’. Those answering, ‘Not worried at all’ or ‘Don’t know’ were classified as ‘Not worried’. Among the 1825 eligible respondents, 66 (3.6%) answered ‘Don’t know’, indicating they are unsure whether they are worried. Since this is a significant proportion, they were not excluded.

Severity

To assess the belief about whether smoking increases COVID-19 severity, respondents were asked: ‘Thinking about people who smoke in general – if people who smoke contracted the coronavirus, how severe do you think the illness would be for them compared to non-people who smoke of the same age?’. Response options included: ‘A lot more severe’, ‘Somewhat more severe’, ‘A little more severe’, ‘Neither more nor less severe’, ‘A little less severe’, ‘Somewhat less severe’, ‘A lot less severe’, ‘Refused’, and ‘Don’t know’. Those who answered ‘A lot more severe’, ‘A little more severe’, or ‘Somewhat more severe’ were classified as ‘More severe’. Those who answered ‘Neither more nor less severe’ were classified as ‘Neither more nor less severe,’ and those who answered ‘A little less severe’, ‘Somewhat less severe’, ‘A lot less severe’, or ‘Don’t know’ were classified as ‘Less severe (not severe)’. Those who chose ‘Refused’ were excluded from the analysis. After COVID-19, the perceived risk of people who smoke regarding general health has increased compared to before^7^, and the increased risk perception of smoking-related diseases is a variable that influences reduced smoking.

*Outcome variable: Reduction in smoking due to COVID-19*

The interplay of perceived susceptibility and severity provides motivation or impetus for action. In contrast, the perception of benefits shapes the preferred action path^22^, potentially influencing changes in smoking behavior during the COVID-19 pandemic. Respondents were asked: ‘What effect has the COVID-19 pandemic had on your smoking?’. Response options were: ‘Because of it, I quit smoking’, ‘Because of it, I’m smoking less’, ‘Because of it, I’m smoking more’, and ‘It has not affected my smoking at all’. Those who answered, ‘No effect’ or indicated ‘Smoking more’ were classified as having ‘No decrease’, whereas those reporting that they smoked less were categorized as ‘Decreased smoking’. Smoking reduction, increase, and no change were categorized as a multinomial variable. However, due to the small number of respondents reporting an increase in smoking, the model fit was not adequate. Therefore, the smoking increases and no change groups were combined into the ‘No decrease’ category for analysis. Supplementary file Table 1 presents the effect of COVID-19 on cigarette smoking among those who smoke or use nicotine products. It shows that there are people who responded that they quit smoking due to COVID-19 despite currently smoking. We believe such responses are inaccurate and would not provide reliable analysis results. Therefore, we excluded those who responded that they quit smoking due to COVID-19 from the analysis.

### Statistical analysis

Unweighted and weighted percentages were used to describe the study sample and Korean population, respectively. All other analyses were conducted using weighted data. An adjusted logistic regression analysis was conducted to explore the association between decreased smoking (vs did not decrease smoking) and increased perceived risk severity. The multivariable model was constructed based on the theoretical framework of the HBM^22^, supported by previous studies showing associations between COVID-19-related risk perceptions and smoking behaviors^5,15,16,18^, as well as empirical relevance observed in preliminary analyses. We examined the interaction effects between the perceived severity of COVID-19 due to smoking and susceptibility, including worry about contracting COVID-19 and developing smoking-related diseases, to determine if susceptibility and severity together influence behavior change, as suggested by the HBM. Additionally, we explored the interaction effects between the perceived severity of COVID-19 due to smoking and modifying factors such as sociodemographic variables and smoking-related variables. Cross-sectional weights were applied, and adjustments were made for stratification and covariates to ensure the estimates were population-representative. All analyses were performed using SAS version 9.4 (SAS Institute, Cary, NC, USA). We used two-tailed tests throughout, and statistical significance was determined at the 0.05 level for both primary associations and interaction analyses. Given the observational and secondary nature of the dataset, formal sample size or power calculations were not applicable.

Ethics approval and consent to participate This study was approved by the Research Ethics Board at the University of Waterloo, Canada (REB#41512), and the Institutional Review Board at Seoul National University, Republic of Korea (IRB No. E2308/002-004). All participants provided informed consent prior to completing the survey.

## RESULTS

Korean adults were much more likely to report reducing their smoking due to the pandemic (18.9%) compared to those who reported increasing their smoking (6.3%) (Table 1). The chi-squared analysis showed a significant difference between the rates of increase and decrease [χ^2^(1)=159.2, p<0.0001]. Meanwhile, 74.8% reported no change in their smoking behavior.

**Table 1:** Sociodemographic and smoking-related characteristics of people aged ≥19 years who smoke only cigarettes in the 2021 ITC Korea Survey (N=1506)

*Characteristics*	*Categories*	*n*	*Unweighted %*	*Weighted %*
**Sex**	Female	152	10.1	7.0
	Male	1354	89.9	93.0
**Age** (years)	19–29	139	9.2	15.1
	30–39	255	16.9	17.3
	40–59	870	57.8	49.9
	≥60	242	16.1	17.7
**Education level**	Low	326	21.6	60.1
	Moderate	1040	69.1	35.5
	High	140	9.3	4.3
**Household annual income**	Low	244	16.2	21.8
	Moderate/high	1262	83.8	78.2
**Marital status**	Not married	560	37.2	46.3
	Married	946	62.8	53.7
**Daily smoking**	No	124	8.2	6.4
	Yes	1382	91.8	93.6
**Heaviness of smoking index**	Low dependence	626	41.6	37.5
	Medium/high dependence	880	58.4	62.5
**Intention to quit**	No	618	41.0	44.0
	Yes	888	59.0	56.0
**Susceptibility**				
**The perceived likelihood of developing smoking-related diseases**	Less likely	270	17.9	18.6
	A little more likely	461	30.6	29.3
	Somewhat more likely	775	51.5	52.1
**Worried about contracting coronavirus**	Not worried	221	14.7	15.0
	A little/somewhat worried	958	63.6	62.3
	Very worried	327	21.7	22.7
**Severity**				
**Perceived severity of COVID-19 due to smoking**	Less severe	264	17.5	19.6
	Neither more nor less severe	484	32.1	31.1
	More severe	758	50.3	49.3
**Decrease smoking due to COVID-19**				
**What effect has the COVID-19 pandemic had on your smoking?**	Decrease	297	19.7	18.9
	Increase	76	5.0	6.3
	No effect at all	1133	75.2	74.8

**Figure 2:**
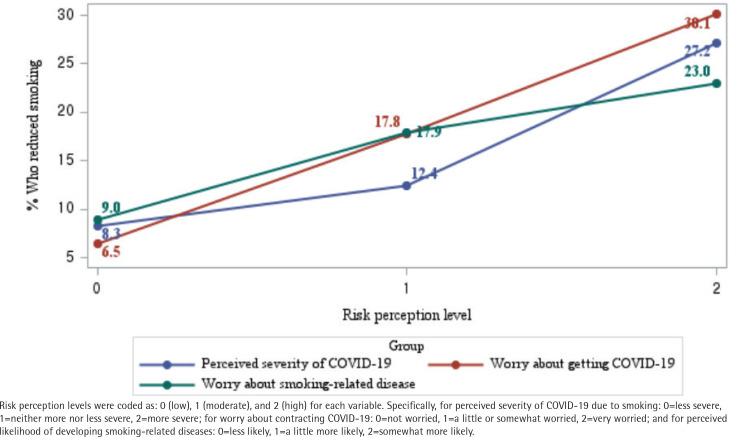
Percentage of people who smoke and reported reducing their smoking due to COVID-19, by levels of perceived COVID-19 severity, worry about getting COVID-19, and worry about smoking-related diseases, based on weighted frequencies from the 2021 ITC Korea Survey (N=1506)

Figure 2 illustrates a consistent increase in smoking reduction rates with higher levels of perceived risk across all three indicators: perceived severity of COVID-19, worry about contracting COVID-19, and concern about smoking-related diseases. Among adults who reported very high levels of worry about contracting COVID-19, 30.1% reduced their smoking, compared to only 6.5% among those with no worry. Similar trends were observed for perceived severity of COVID-19 and concern about smoking-related diseases.

Table 2 describes the results of the adjusted regression analyses. We found that adults who were extremely/very worried about contracting COVID-19 were significantly more likely to have reduced their smoking (AOR=4.25; 95% CI: 1.65–10.99) compared to those who were not worried. Adults who believe that smoking makes COVID-19 more severe were significantly more likely to have reduced their smoking (AOR=2.34; 95% CI: 1.19–4.61) than those with less perceived. We did not find a significant association between worry of getting a smoking-related disease and a reduction in smoking (AOR=1.27; 95% CI: 0.60–2.72; AOR=1.46; 95% CI: 0.72–2.94). Individuals aged ≥60 years, moderate to high household income, and having intentions to quit were significantly more likely to have reduced their smoking (AOR=2.60; 95% CI: 1.12–6.03; AOR=2.53; 95% CI: 1.34–4.76; AOR=5.26; 95% CI: 3.21–8.59), while those who smoked daily and those with medium or high nicotine dependence were less likely to reduce their smoking (AOR=0.36; 95% CI: 0.18–0.71; AOR=0.60; 95% CI: 0.39–0.92).

**Table 2:** Adjusted logistic regression results on factors associated with smoking reduction due to COVID-19 among people who smoke only cigarettes in Korea (N=1506)

*Variables*	*Categories*	*Decreased smoking* *(Ref. no decrease)*	
		*AOR*	*95% CI*
**Sociodemographic variables**			
**Age** (years)	19–29 ®	1	
	30–39	1.03	0.51–2.06
	40–59	1.03	0.50–2.11
	≥60	2.60*	1.12–6.03
**Sex**	Female ®	1	
	Male	1.09	0.55–2.18
**Education level**	Low ®	1	
	Middle	0.82	0.56–1.22
	High	0.81	0.42–1.58
**Household annual income^a^**	Low ®	1	
	Moderate/high	2.53*	1.34–4.76
**Marital status**	Not married ®	1	
	Married	0.70	0.40–1.24
**Smoking-related variables**			
**Daily smoking**	Non-daily ®	1	
	Daily	0.36*	0.18–0.71
**Heaviness of Smoking Index**	Low dependence ®	1	
	Medium/high dependence	0.60*	0.39–0.92
**Intention to quit smoking**	No ®	1	
	Yes	5.26*	3.21–8.59
**Susceptibility**			
**The perceived likelihood of developing smoking-related diseases**	Less likely ®	1	
	A little more likely	1.27	0.60–2.72
	Somewhat more likely	1.46	0.72–2.94
**Worried about contracting coronavirus**	Not worried ®	1	
	A little/somewhat worried	2.40	0.96–5.97
	Very worried	4.25*	1.65–10.99
**Severity**			
**Perceived severity of COVID-19 due to smoking**	Less severe ®	1	
	Neither more nor less severe	1.43	0.66–3.13
	More severe	2.34*	1.19–4.61

Results from a binary logistic regression model are presented. The number of observations used is 1506, with complete data for all variables. Data are weighted. AOR: adjusted odds ratio; adjusted for age, sex, education level, marital status, household income, daily smoking, perceived likelihood of developing smoking-related diseases, Heaviness of Smoking Index, intention to quit smoking, worrying about getting coronavirus.

a Household annual income in million KRW (low: <30, moderate: 30 to <75, high: ≥75). KRW: 1000 Korean Won about US$0.73. ® Reference categories.

* Significant at the p<0.05 level.

**Figure 3:**
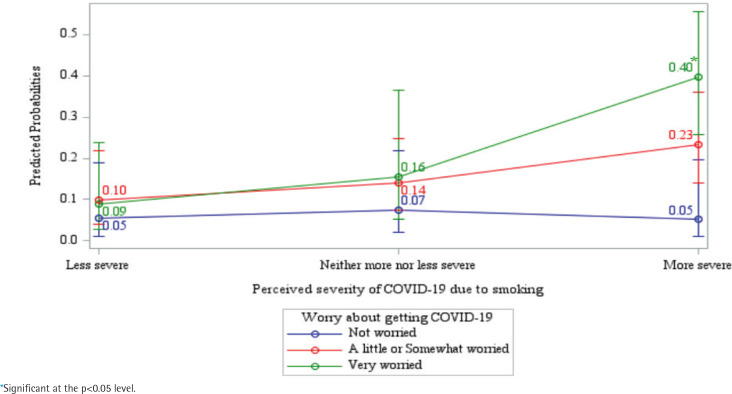
Predicted probabilities of smoking reduction by perceived COVID-19 severity and worry about getting COVID-19: Interaction analysis among Korean adults who smoke only cigarettes 2021 ITC Korea Survey (N=1506)

Table 3 shows a significant interaction between the belief that smoking worsens COVID-19 severity and the worry about contracting COVID-19 in predicting smoking reduction (B=2.41, p=0.049). The predicted probability of smoking reduction was approximately 40% among participants who were very worried about contracting COVID-19 and believed that smoking increases its severity, compared to significantly lower probabilities in other groups, such as those who did not worry about infection and perceived smoking as less severe in relation to COVID-19. Figure 3 presents the specific pattern of the interaction. Among those who smoke who do not believe that smoking makes COVID-19 more severe and worry about getting COVID-19, does not predict smoking reduction. It is only for those who do believe that smoking makes COVID-19 more severe that worry about getting COVID-19 is predictive of smoking reduction – since such individuals perceive that their smoking has consequences, and that is translated into action to reduce the threat.

**Table 3:** Interaction effects between perceived COVID-19 severity due to smoking and sociodemographic, behavioral, and risk perception variables on smoking reduction among Korean adults (N=1506)

*Variables*	*Categories*	*Perceived severity of COVID-19 due to smoking (Ref. less severe)*					
		*Neither more nor less severe*			*More severe*		
		*Estimate*	*SE*	*p*	*Estimate*	*SE*	*p*
**Sociodemographic variables**							
**Age** (years)	19–29 ®						
	30–39	0.30	1.21	0.80	0.01	1.10	1.00
	40–59	-0.77	1.13	0.50	-0.65	1.03	0.53
	≥60	1.33	1.24	0.28	0.91	1.11	0.41
**Sex**	Female ®						
	Male	-0.49	0.98	0.62	-0.98	0.81	0.23
**Education level**	Low ®						
	Moderate	-0.94	0.74	0.20	-0.94	0.66	0.16
	High	0.46	1.52	0.76	-0.33	1.43	0.82
**Household annual income**	Low ®						
	Moderate/high	-0.57	1.11	0.61	-0.13	0.96	0.89
**Marital status**	Not married ®						
	Married	0.82	0.77	0.29	0.62	0.69	0.37
**Smoking-related variables**							
**Daily smoking**	Non-daily ®						
	Daily	-0.95	1.20	0.43	-0.25	1.04	0.81
**The Heaviness of Smoking Index**	Low dependence ®	-0.72	0.75	0.34	0.01	0.68	0.99
	Medium/high dependence						
**Intention to quit smoking**	No ®						
	Yes	-0.56	0.74	0.45	-0.52	0.67	0.44
**Susceptibility**							
**Worry about getting coronavirus**	Not worried ®						
	A little/ somewhat worried	-0.46	1.05	0.66	1.14	1.16	0.33
	Extremely/very worried	0.13	1.23	0.92	2.41*	1.23	0.05
**The perceived likelihood of developing smoking-related diseases**	Less/just as likely ®						
	A little/ somewhat more likely	-0.12	1.07	0.91	0.00	1.04	1.00
	Much more likely	0.73	1.01	0.47	1.12	0.97	0.25

Estimate values are unstandardized regression coefficients. SE: standard error. ® Reference categories.

* Significant at the p<0.05 level.

## DISCUSSION

One in five Koreans who smoked cigarettes exclusively in 2021 reduced their smoking due to COVID-19, a rate over three times higher than those who reported increasing their smoking. COVID-19 perceived susceptibility and severity increased independently with smoking reduction and when the two factors interacted. In contrast, worrying about smoking-related diseases did not reduce smoking. Being older (aged ≥60 years), having a middle or high income, and intentions to quit were associated with decrease in smoking. People who smoke daily and those with moderate or high nicotine dependence were less likely to cut back on smoking.

Two COVID-19-related measures – worry about contracting COVID-19 and greater perceived COVID-19 severity for people who smoke – predicted reductions in smoking, both independently and interactively. Despite a lack of evidence that smoking increases susceptibility to SARS-CoV-2 infection, people who smoke may not distinguish between the risks of susceptibility and severity. Statements from WHO and other health authorities identifying people who smoke as a high-risk group made it reasonable to assume that both HBM constructs were responsible for that high risk. Our findings are similar to a previous study from the early phase of the COVID-19 pandemic, and included four westernized countries, which showed that fears of contracting COVID-19 and perceptions of severe COVID-19 influenced smoking reduction^5^.

Unlike the previous study conducted in 2020^5^, our 2021 study did not find that being slightly or somewhat worried about contracting COVID-19 reduced smoking. This discrepancy may be due to a better understanding over time that smoking is not related to contracting COVID-19 but is associated with the severity of the disease. Unlike previous studies that analyzed people using both other nicotine products and smoking cigarettes^5^, this study is significant as it focuses on people who smoke only cigarettes, demonstrating a reduction in cigarette consumption. Conducted in 2021, it shows that the association between smoking reduction and these variables persisted beyond the first year of the pandemic.

Contrary to our study, a US study conducted in early 2020 found that perceived risk during COVID-19 was associated with motivation to quit but not with changes in usage^6^. Additionally, most participants did not change their usage after becoming aware of COVID-19, and more people increased their usage than reduced it^6^. Previous studies have shown that people who smoke often underestimate the health risks associated with smoking, and this may also apply to the risks of COVID-19 ^23^.

In this study, people aged ≥60 years reduced smoking during COVID-19 more than those in their 20s. The severity and symptoms of COVID-19 infection are known to increase with age^24^. Additionally, among people aged 50–66 years, functional impairment has been identified as the strongest predictor of smoking cessation, suggesting that these people may have reduced smoking to take care of their health^25^. We also found that smoking reduction was more pronounced among individuals with higher household income. People with lower socioeconomic status may have lower intentions to quit smoking due to lower levels of education and less concern about the harmful effects of smoking^26^. Additionally, the COVID-19 pandemic has exacerbated health inequalities among vulnerable populations, closely linked to lower levels of education, inadequate knowledge, and improper preventive behaviors^27^.

Our study showed that intention to quit smoking was associated with smoking reduction due to COVID-19. While not surprising, previous studies indicate that despite heightened intentions^6^, or even thoughts about quitting^5^, most did not change their smoking during the pandemic. Similarly, other studies suggested that intentions do not consistently lead to actual behavior changes, reflecting the ‘intention-behavior gap’^28^. However, intentions to quit are crucial for reducing smoking and preparing for cessation. According to planned behavior theory, this intention to quit is a decisive element in smoking reduction^29^. Therefore, this study’s finding that intention to quit influenced smoking reduction aligns with previous theoretical frameworks.

People with medium or high nicotine dependence, or who smoke daily were less likely to reduce smoking during COVID-19. Previous studies show that people who smoke less frequently are more likely to express a desire to quit smoking and succeed compared to people who smoke daily^30^. People who smoke occasionally tend to have lower nicotine dependence than people who smoke daily^31^. Thus, healthcare providers should systematically identify people with high nicotine dependence and encourage them to stop smoking and offer interventions and coping strategies to encourage quit attempts and maintain abstinence after quitting.

In other countries, reductions in smoking due to COVID-19 were often not greater than increases^4-6^, but in Korea, the strong COVID-19 response and extensive information about the impact of smoking on COVID-19 severity may have played a crucial role in increasing risk perception and encouraging people who smoke to reduce their smoking. The findings on worry about COVID-19 infection and perceived severity influencing smoking reduction, support campaigns and education on smoking risks during respiratory infectious diseases. Previous studies reported that messages from the US Centers for Disease Control and Prevention’s (CDC) X (formerly Twitter)^32^ and infographics from the Korean Ministry of Health and Welfare increased risk perceptions about smoking and severe COVID-19 outcomes, as well as the infection risks associated with smoking behaviors like removing masks or touching the mouth^19^. This heightened risk perception among people who smoke suggests the pandemic provided an opportunity to reduce smoking rates^32^.

Immediate threats, such as COVID-19 infection or severe illness, rather than future risks like smoking-related diseases, were associated with reducing smoking. This indicates that immediate threats are stronger motivators for reducing cigarette consumption or quitting altogether^33^. People tend to discount future outcomes and prefer short-term results, which makes worry about smoking-related diseases less motivating for smoking reduction^34^. Loss framing in health messages can increase perceived susceptibility and risk relevance, enhancing the intention to quit smoking.

Many people who smoke recognize the health risks but often underestimate their own susceptibility, believing others are more at risk^35^. Emphasizing losses through negative framing may be less effective due to self-enhancing bias, where people view themselves more positively than in reality. Positively framed messages highlighting the benefits of quitting smoking and enhancing self-efficacy could be more effective in driving behavior change. Highlighting the advantages of quitting smoking can potentially drive behavior change more effectively. Additionally, including information in these messages that communicates and enhances self-efficacy for people who smoke could be significant, encouraging them to believe that they can successfully reduce smoking^36^.

This study focused on Koreans who smoked only cigarettes to investigate changes in cigarette (nicotine) consumption precisely. Including Koreans who used other nicotine products in the analysis could show a reduction in cigarette smoking due to switching to other nicotine products, thus confounding evidence of a decrease in cigarette consumption.

### Limitations

One limitation of this study was its small sample size, resulting from examining the effect of reduced smoking among those who smoked only cigarettes. The second limitation is that this study focused on a decrease in smoking quantity rather than assessing the cessation. In addition, since the study population was limited to Korean adults, the findings may not be generalizable to populations in other countries with different tobacco control contexts or public health communication strategies. The cross-sectional and observational nature of the data also precludes causal inferences. Although multiple covariates were adjusted for, the possibility of residual confounding cannot be ruled out.

## CONCLUSIONS

One in five Korean adults reduced their smoking due to COVID-19, a rate that is three times higher than those who increased their smoking. This study found that concerns about smoking-related disease did not reduce smoking, but greater worry about contracting COVID-19 and the belief that smoking would worsen its severity did. This suggests that immediate threats may have a greater impact on reducing smoking than distant threats. Additionally, there is a need for policies or campaigns targeting people who smoke daily and those with nicotine dependence.

## Supplementary Material



## Data Availability

The data supporting this research are available from https://itcproject.org/request-data-form/ In each country participating in the International Tobacco Control Policy Evaluation (ITC) Project, the data are jointly owned by the lead researcher(s) in that country and the ITC Project at the University of Waterloo. Data from the ITC Project are available to approved researchers two years after the date of issuance of cleaned data sets by the ITC Data Management Centre. Researchers interested in using ITC data are required to apply for approval by submitting an International Tobacco Control Data Repository (ITCDR) request application and subsequently signing an ITCDR Data Usage Agreement. The criteria for data usage approval and the contents of the Data Usage Agreement are described online (http://www.itcproject.org).
