# Supplementary material for: An examination of the relationship between perceptions that cigarette smoking increases the severity of COVID-19 and reduction in smoking during the COVID-19 pandemic: Findings from the 2021 ITC Korea Survey
Source: Tob Induc Dis. 2025 Jun 30;23:10.18332/tid/205468. doi: 10.18332/tid/205468 (PMC12215669; doi:10.18332/tid/205468)
Supplement: Supplementary file 1 [file TID-23-88-s1.pdf]

Supplementary File

Supplemental Figure 1. Study Flow Diagram of participant eligibility and sample selection in the 2021 International Tobacco Control (ITC) Korea Survey (N=4,467)

All respondents who completed the ITC Korea Wave 2 (2021) Survey
n=4,467

Total respondents excluded, n=2,642

Using heated tobacco products exclusively: 153
Using electronic cigarettes exclusively: 67
Using heated tobacco products and cigarettes:1,123
Using electronic cigarettes and cigarettes:165
Using heated tobacco products and electronic cigarettes: 44
Using heated tobacco products, electronic cigarettes, and cigarettes: 444
Recently quit using heated tobacco products but not electronic cigarettes: 8
Recently quit* using electronic cigarettes but not heated tobacco products: 14
Recently quit* using both heated tobacco products and electronic cigarettes: 2
Recently quit* not using heated tobacco products and electronic cigarettes: 60
Did not use any product and quit >2 years**: 562

*Respondents who had quit smoking cigarettes in the last 2 years but had smoked at least 100 cigarettes in their lifetime.
**Those who quit smoking cigarettes >2 years at Wave 2.

Eligible for study inclusion, n=1,825

Respondents who smoked cigarettes exclusively

Total respondents excluded, n=319

Respondents who did not answer questions regarding perception, outcome and covariate variables.

Eligible for study inclusion, n=1,506

Respondents who smoked cigarettes exclusively and answered questions regarding perception, outcome variable, and covariate variables.

Supplementary Table 1

Self-reported changes in cigarette smoking due to COVID-19 by type of nicotine product used among Korean adults in the 2021 ITC Korea Survey (N=4,467)

Effect of the outbreak on smoking	Type of nicotine products used*												
	excl Cigs	excl HTPs	excl ECs	Cigs +HTPs	Cigs +ECs	HTPs +ECs	Triple products	Quit within 2y using HTPs, but not ECs	Quit within 2y using ECs, but not HTPs	Quit within 2y and using both ECs and HTPs	Quit within 2y not using either HTPs or ECs	Not using any products	Total
Because of it, I quit smoking.	38	3	2	19	2	7	26	0	0	0	5	13	115
Because of it, I'm smoking less.	318	32	15	264	27	14	155	3	0	0	4	28	860
Because of it, I'm smoking more.	85	13	4	58	16	2	60	0	2	0	0	8	248
It has not effect at all on smoking	1248	97	45	708	106	18	181	5	11	2	44	369	2834
Refused	6	0	0	5	1	0	3	0	0	0	0	34	49
Don't know	130	8	1	69	13	3	19	0	1	0	7	110	361
Total	1825	153	67	1123	165	44	444	8	14	2	60	562	4467

*Note: excl (exclusive), Cigs (cigarettes), HTP (Heated tobacco products), ECs (Electronic cigarettes)

Supplementary table 2 Interaction effects between perceived COVID-19 severity due to smoking and sociodemographic, behavioral, and risk perception variables on smoking reduction among Korean adults (N=1,506)

Perceived severity of COVID-19 due to smoking (reference= Less severe)			Neither more nor less severe			More severe		
			Estimate	SE	P	Estimate	SE	P
Socio-demographic variables	Age	19-29 years old	Reference					
		30-39 years old	0.30	1.21	0.80	0.01	1.10	1.00
		40-59 years old	-0.77	1.13	0.50	-0.65	1.03	0.53
		60+ years old	1.33	1.24	0.28	0.91	1.11	0.41
	Sex	Female	Reference					
		Male	-0.49	0.98	0.62	-0.98	0.81	0.23
	Educational level	Low	Reference					
		Moderate	-0.94	0.74	0.20	-0.94	0.66	0.16
		High	0.46	1.52	0.76	-0.33	1.43	0.82
	Household income	Low	Reference					
		Moderate + High	-0.57	1.11	0.61	-0.13	0.96	0.89
	Marital status	Not married	Reference					
		Married	0.82	0.77	0.29	0.62	0.69	0.37
Smoking-related variables	Daily smoking	Non-daily	Reference					
		Daily	-0.95	1.20	0.43	-0.25	1.04	0.81
	The Heaviness of Smoking Index	Low dependence	Reference					
		Medium+High dependence	-0.72	0.75	0.34	0.01	0.68	0.99
	Intention to quit smoking	No	Reference					
		Yes	-0.56	0.74	0.45	-0.52	0.67	0.44
Suscepti-bility	Worry about getting coronavirus	Not worried	Reference					
		A little or Somewhat worried	-0.46	1.05	0.66	1.14	1.16	0.33
		Extremely or very worried	0.13	1.23	0.92	2.41*	1.23	0.05
	The perceived likelihood of developing smoking-related diseases	Less or Just as likely	Reference					
		A little or somewhat more likely	-0.12	1.07	0.91	0.00	1.04	1.00
		Much more likely	0.73	1.01	0.47	1.12	0.97	0.25

Note: Estimate values are unstandardized regression coefficients, *p<0.05, Standard Error(SE)

© 2025 Chun H.R. et al.
